# Identifying patients with medically unexplained physical symptoms in electronic medical records in primary care: a validation study

**DOI:** 10.1186/1471-2296-15-109

**Published:** 2014-06-05

**Authors:** Madelon den Boeft, Johannes C van der Wouden, Trudie R Rydell-Lexmond, Niek J de Wit, Henriëtte E van der Horst, Mattijs E Numans

**Affiliations:** 1 Department of General Practice and Elderly Care Medicine, EMGO Institute for Health and Care Research, VU University Medical Center, Van der Boechorststraat 7, 1081 BT, Amsterdam, The Netherlands; 2 Department of General Practices, Julius Center for Health Sciences and Primary Care, University Medical Center Utrecht, Universiteitsweg 100, 3584 CG, Utrecht, The Netherlands; 3 Department of Public Health and Primary Care LUMC, Albinusdreef 2, 2333 ZA, Leiden, The Netherlands

## Abstract

**Background:**

When medically unexplained physical symptoms (MUPS) become persistent, it may have major implications for the patient, the general practitioner (GP) and for society.

Early identification of patients with MUPS in electronic medical records (EMRs) might contribute to prevention of persistent MUPS by creating awareness among GPs and providing an opportunity to start stepped care management. However, procedures for identification of patients with MUPS in EMRs are not well established yet. In this validation study we explore the test characteristics of an EMR screening method to identify patients with MUPS.

**Methods:**

The EMR screening method consists of three steps. First, all patients ≥18 years were included when they had five or more contacts in the last 12 months. Second, patients with known chronic conditions were excluded. Finally, patients were included with a MUPS syndrome or when they had three or more complaints suggestive for MUPS. We compared the results of the EMR screening method with scores on the Patient Health Questionnaire-15 (PHQ-15), which we used as reference test. We calculated test characteristics for various cut-off points.

**Results:**

From the 1223 patients in our dataset who completed the PHQ-15, 609 (49/8%) scored ≥5 on the PHQ-15. The EMR screening method detected 131/1223 (10.7%) as patients with MUPS. Of those, 102 (77.9%) scored ≥5 on the PHQ-15 and 53 (40.5%) scored ≥10. When compared with the PHQ-15 cut-off point ≥10, sensitivity and specificity were 0.30 and 0.93 and positive and negative predictive values were 0.40 and 0.89, respectively.

**Conclusions:**

The EMR screening method to identify patients with MUPS has a high specificity. However, many potential MUPS patients will be missed. Before using this method as a screening instrument for selecting patients who might benefit from structured care, its sensitivity needs to be improved while maintaining its specificity.

## Background

Presentation of medically unexplained physical symptoms (MUPS) is a common phenomenon in primary care. Of all primary care encounters, in up to a third the symptoms presented by the patient remain unexplained [1,2]. In specialist care, these figures may even be higher, depending on the specialty [3]. Although MUPS become persistent in only a minority (2.5%) of patients, the burden of persistent MUPS is high for both patients and doctors and for society [4]. Patients are functionally impaired and may feel that they are not taken seriously by their general practitioner (GP) [5-7]. Furthermore, the doctor-patient relationship is often troubled and many GPs indicate that they find these patients difficult to manage [8,9]. Also persistent MUPS may lead to high and inadequate health care utilization and high associated costs [10-12].

Early identification of patients with a higher risk of developing persistent MUPS in routine electronic medical records (EMRs) could create an opportunity for proactive and structured care, taking into account the severity of MUPS, coordinated by GPs. Awareness among GPs of their population at risk could result in more attention during consultations or in offering effective interventions like cognitive behaviour therapy at an earlier stage if appropriate [13]. The advantage of using EMRs is that the data are directly available and no additional data collection is needed, which saves time consuming logistical procedures. Furthermore it provides a quick overview of a population at risk.

Early identification in EMRs proved to be feasible and effective for other risk populations, like patients with type 2 diabetes, cardiovascular risks and frail elderly [14-16] as well as for preventive health care [17]. Also Tian et al. developed an applicable EMR algorithm to identify patients with chronic pain [18]. However, identifying patients with MUPS is not an easy task as there is no generally accepted procedure available. Although some MUPS characteristics, like frequent consultation and referral rate, can be obtained from EMRs, there is no *international classification of primary care* (ICPC) code available that identifies the combination of symptoms that characterise MUPS of various MUPS subgroups.

Morriss et al. developed an EMR model that estimates the prevalence of MUPS. However, they concluded that the model is not useful for screening purposes due to a low sensitivity [19]. Various other methods for MUPS screening have been developed and studied. Kroenke et al. showed in their validation study that the self-administered Patient Health Questionnaire-15 (PHQ-15) could be used for screening somatisation and somatic symptom severity including MUPS [20]. However, the PHQ-15 can not be easily obtained from EMRs. Verhaak et al. used criteria composed by Robbins et al. to estimate the prevalence of persistent MUPS, but in their study it is about the patients who already suffer from persistent MUPS and not about the patients at risk [4,21].

In 2010, a cross-sectional study focusing on the prevalence of MUPS was conducted in the Utrecht Health Project. Patients with MUPS were identified using EMR data in three subsequent selection steps. In our current study we aim to validate this EMR screening method to identify MUPS patients by comparing it to the commonly used and validated PHQ-15.

## Methods

### Setting and study population

The Utrecht Health Project is a primary care population study with the purpose to enable research into the impact of changes in health care policy, developments in public health and quality management, as well as to support population research into determinants of health and disease [22]. From 2000 on, all inhabitants of the new neighbourhood Leidsche Rijn near Utrecht enlisted with local GPs were invited to participate. Various health measurements and questionnaires were collected after informed consent, including the PHQ-15 since 2005, and they were linked with follow-up data extracted from EMRs. For our current study we were able to use EMRs from 1223 patients 18 years or older who completed the PHQ-15 in 2005–2007. We retrieved EMR data for each patient over the 12 months preceding the completion of the PHQ-15.

### Study design and procedure

In this validation study we compared the results of the EMR screening method for detecting possible MUPS patients with the PHQ-15 scores of the patients as a reference standard. We defined patients with scores from cut-off point ≥5 on the PHQ-15 as having MUPS, following Kroenke et al. [20].

### The EMR screening method

The EMR screening method identifies patients with possible MUPS with three subsequent selection steps. First step: Patients ≥18 years with ≥5 general practice consultations during the past 12 months were selected, as it is known that MUPS patients usually have relatively high consultation rates [11]. Second step: patients with chronic obstructive pulmonary disease, hypertension or diabetes mellitus and patients with an established psychiatric diagnosis were excluded, in order to exclude all patients in whom physical symptoms are medically explained and because frequent practice visits might at forehand be assumed for these conditions [23,24].

Third step: In the remaining group, patients were selected who consulted the GP with one of the three MUPS syndromes; irritable bowel syndrome (ICPC D93), fibromyalgia (ICPC L18.01) and chronic fatigue syndrome (ICPC A04.01). This group was called “Syndrome-based Confirmed-MUPS”. Furthermore we selected all patients who had three or more contacts with at least one of a list of 104 ICPC codes suggestive of MUPS, as assessed by the GPs during regular care (symptom diagnoses) (Additional file 1). This group was called “High-risk-MUPS”. “High-risk-MUPS” and “Syndrome-based Confirmed MUPS” together formed the complete MUPS risk population resulting from these selection steps.

### Patient health questionnaire-15

**Figure 1:**
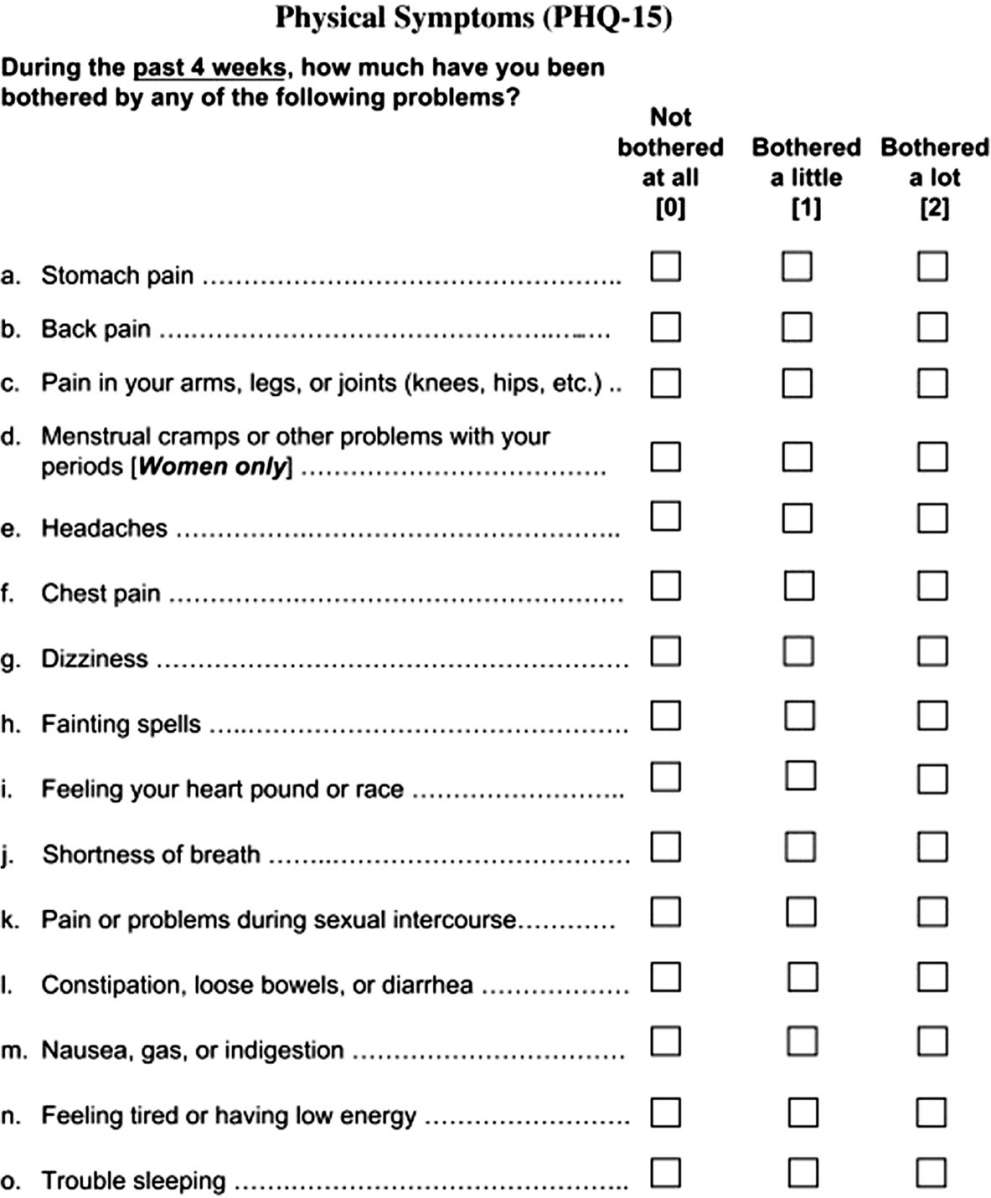
Patient Health Questionnaire-15.

Internationally, the PHQ-15 (Figure 1) is a widely used and validated mental health screening instrument to assess the severity of somatic symptoms. It is based on the “Primary Care Evaluation of Mental Disorders” (PRIME-MD), a diagnostic instrument for common mental health disorders and the “PRIME-MD Patient Health Questionnaire” [25,26]. It inquires into 15 symptoms or symptoms clusters that account for more than 90 percent of physical complaints. Thirteen somatic symptoms of the PRIME-MD are included in the PHQ-15 and two symptoms are part of the PHQ depression module. For each item, there are three options to score in severity of complaints; zero (not bothered at all), one (bothered a little) and two (bothered a lot), resulting in overall score ranging from zero to 30. For all symptoms, a score of two is considered severe. In two studies it was concluded that the PHQ-15 is a valid and moderately reliable questionnaire that may be used to detect MUPS. Therefore we used this questionnaire as the reference method in our study [20,27].

### Ethical approval

All participants in the Utrecht Health Project gave informed consent for linking their anonymous EMRs to the PHQ-15. The medical ethical committee of University Medical Center Utrecht approved the original protocol of the Utrecht Health Project and its amendments (file#99-240). The current study made use of readily available data and did not require additional informed consent or ethical approval.

### Statistical analysis

In our dataset consisting of 1223 patients who completed the PHQ-15, we calculated the prevalence of MUPS patients following the EMR screening method. We dichotomized the continuous PHQ-15 outcome by using two cut-off points for mild and medium MUPS; ≥5 and ≥10 respectively, as used by Kroenke et al. [20]. Less than 2% of all entries were missing. Missing data in the PHQ-15 were imputed. Sensitivity analysis showed only minor differences between complete and imputed cases. The multiple imputation model included age, gender, total number of contacts, all 15 PHQ-15 questions and the outcome variable MUPS. Crosstabulations enabled us to calculate sensitivity, specificity, predictive values and likelihood ratios for the two cut-off points including 95% confidence intervals. All analyses were processed with SPSS version 20.0.

## Results

### Prevalence of MUPS

We assessed the prevalence of the MUPS risk population in our dataset of 1223 adult patients, consisting of 756 women (61.8%) and 467 men (38.2%) by carrying out the described steps. The mean age was 38.8 years. Twenty-one patients (1.7%) were identified as “Confirmed MUPS”. All 21 were diagnosed with irritable bowel syndrome, for which they had had at least one consultation in the 12 months period. There were no patients with an ICPC code for chronic fatigue syndrome or fibromyalgia. The EMR screening method identified 126 patients (10.3%) as “High-risk-MUPS”. Most patients with irritable bowel syndrome also had at least one ICPC code suggestive of MUPS. Together, the total MUPS prevalence of both groups combined in this population according to the EMR method was 131 (10.7%). Of those, 93 (71%) were women, significantly more than men (p = 0.04).

### PHQ-15 outcomes

In the total population, 609 patients (49.8%) scored ≥5 on the PHQ-15 and 176 (14.4%) ≥10. The PHQ-15 results were skewed (skewness 1.27; Kolmogorov-Smirnov p < 0.001) with a mean of 5.29 and a median of 4.0. In the MUPS group selected by the EMR screening method, 102/131 (77.9%) patients scored ≥5 on the PHQ-15 and 53/131 (40.5%) scored ≥10. Again, the distribution was skewed (skewness 0.51; Kolmogorov-Smirnov P < 0.001) with a mean and median of 8.57 and 8.0, respectively. Of all 21 patients with at least one contact for irritable bowel syndrome, 19 (90.5%) scored ≥5 on the PHQ-15 and 13 (61,9%) scored ≥10.

### Test characteristics of the EMR screening method compared with the PHQ-15

For cut-off point ≥5, sensitivity and specificity of the EMR screening method were 0.17 and 0.95, respectively. The likelihood ratios for a positive and negative test were 3.54 and 0.87, respectively. Positive and negative predictive values were 78% and 54%, respectively. For the cut-off point ≥10, sensitivity and specificity were 0.30 and 0.93, respectively. The likelihood ratio for a positive test was 4.29, for a negative test 0.75 and positive and negative predictive values were 40% and 89%, respectively (Tables 1, 2, and 3).

**Table 1:** **Two**-**by**-**two table of PHQ**-**15 cut**-**off point 5**

	**PHQ-15 ≥ 5**	**PHQ-15 < 5**	**Total**
**EMR screening method ‘MUPS’**	102	29	131
**EMR screening method ‘no MUPS’**	507	585	1092
**Total**	609	614	1223

**Table 2:** **Two**-**by**-**two table of PHQ**-**15 cut**-**off point 10**

	**PHQ-15 ≥ 10**	**PHQ-15 < 10**	**Total**
**EMR screening method ‘MUPS’**	53	78	131
**EMR screening method ‘no MUPS’**	123	969	1092
**Total**	176	1047	1223

**Table 3:** **Comparing the EMR screening method with the PHQ**-**15 cut**-**off scores**

	**PHQ-15** ≥ **5 (95% confidence interval)**	**PHQ-15** ≥ **10 (95% confidence interval)**
Sensitivity	0.17 (0.14 - 0.20)	0.30 (0.24 – 0.38)
Specificity	0.95 (0.93 - 0.97)	0.93 (0.91 – 0.95)
Positive predictive value	0.78 (0.71 – 0.85)	0.40 (0.32 – 0.49)
Negative predictive value	0.54 (0.51 – 0.57)	0.89 (0.87 – 0.91)
Likelihood ratio positive test	3.54 (2.38 – 5.27)	4.29 (2.96 – 5.51)
Likelihood ratio negative test	0.87 (0.84 – 0.91)	0.75 (0.69 – 0.83)

## Discussion

### Main findings

The aim of our study was to validate the EMR screening method to identify MUPS patients using the PHQ-15 as a reference test in order to map a specific and heterogeneous population at risk that might benefit from structured and stepped care. We found a prevalence of 10.7% with the EMR screening method compared to a high prevalence of 49.8% with the PHQ-15 cut-off ≥5. Most MUPS patients identified by the EMR screening method and patients with IBS scored at least 5 points on the PHQ-15. Test characteristics showed a high specificity but a low sensitivity for both PHQ cut-off points, which indicates that about 80% of patients with MUPS were missed.

### Interpretation of results

The prevalence of MUPS has been frequently studied and varies greatly. In most studies, percentages range around 30 percent in primary care [1,5,28]. In our study, almost half of all patients scored positive on the PHQ-15 cut-off ≥5, suggesting that many patients in this group of patients probably have incidental complaints and will not benefit from proactive care. The prevalence of 10.7% found by the EMR screening method is lower. The main reason for the difference between our results and existing literature seems to be that the EMR screening method is rather stringent. Various other reasons can also account for the difference. First, the quality of registration in the participating practices may be suboptimal. In this study, only 21 (1.7%) patients were found with an ICPC code for irritable bowel syndrome, a much lower prevalence than what is known from research, namely 14 to 24 percent of women and five to 19 percent of men [29]. However, our findings are consistent with the results from other Dutch studies in routine healthcare data [30]. We did not find patients with a coded diagnosis of chronic fatigue syndrome or fibromyalgia, where one or two could be expected [31]. Patients with chronic fatigue or chronic widespread pain, closely related to fibromyalgia, might have been recorded with other diagnostic terms than L18.01 (fibromyalgia) or A04.01 (chronic fatigue syndrome). These patients, however, will be found in the third step of our selection where MUPS suggestive codes are selected, such as fatigue (A04), general pain (A01) and muscle pain (L18).

Second, we only considered ICPC codes registered during the year preceding the patients’ PHQ-15 score. We did not include patients with a MUPS suggestive or MUPS syndrome ICPC code registered before that time which could have resulted in false negatives. Finally, all patients with known chronic somatic or psychiatric comorbidity were excluded, while studies show that especially those patients more often suffer from unexplained symptoms [23,24].

The high specificity but low sensitivity can partly be explained by the fact that patients do not present all symptoms to GPs and GPs do not code all presented symptoms in their EMRs. Furthermore, the doctor’s diagnostic label is a reflection of the symptom the patient presented and it is his understanding of the situation.

### Strengths and limitations

Because the only selection criteria of our research population were if they lived in a certain area (Leidsche Rijn) and completed the PHQ-15, we minimized selection bias and response tendencies. We had more women than men in our study because women completed the PHQ-15 more often than men. This is consistent with gender differences in the number of GP encounters in the Netherlands. We also found a significant difference between men and women in the prevalence of MUPS which is also consistent with other studies [32,33].

Three study limitations should be noted. The first is that no gold standard is available for defining a ‘true’ MUPS population. In the end, only the physician decides whether the patients’ symptoms are medically explained or not, entailing a certain amount of subjectivity. We chose to use the PHQ-15 because of its availability and as a second best reference standard after the physician’s judgement as this self-administered questionnaire has been validated for clinical practice and research for screening and monitoring MUPS and somatoform disorders by Kroenke and van Ravensteijn [20,27]. Kroenke et al. concluded that high total scores strongly correlate with distress, functional impairment and with increased healthcare use, which supports our choice of the PHQ-15 as a reference standard. However, Kroenke et al. noted that the PHQ-15 could not completely replace the GPs clinical judgment as it cannot distinguish between explained and unexplained symptoms. “Also, obviously using the PHQ-15 as the primary instrument to find MUPS in primary care should not be advised because of the high percentage found when using cut-off point 5 or more”.

Second, when registration by practice employees is not complete and uniform according to existing guidelines and therefore suboptimal, the performance of any EMR search strategy will hamper. Third, MUPS are often associated with frequent attendance, but not always, particularly not in the early stages. By identifying patients with at least 5 preceding consultations, some patients with MUPS in the earlier stages might be missed.

### Implications for research and clinical practice

An accurate screening method for retrieving data from EMRs has many advantages for research or care purposes. The identified population can be offered to GPs who should judge if their patients have MUPS or not and should consider proactive and structured stepped care management, depending on the severity of MUPS, for example with panel management to prevent persistence [34,35]. Looking specifically at this EMR screening method, increasing the sensitivity while maintaining the level of specificity will make it more suitable for proactive panel management. “Potential improvement might be reached with the addition of prescription of analgesics or opiate drugs in patient groups without relevant comorbidity as a predictor. Smits et al. have demonstrated that this kind of prescription is associated with frequent attendance [36,37]. However, in our relatively small study population relatively few opiate drugs were prescribed without underlying malignancy and many analgesics are freely available and therefore not registered, so we were not able to include prescription of analgesics in our analysis reliably”.

## Conclusion

Early identification of MUPS patients in EMRs might support GPs to structure care and to initiate proactive stepped care management. The assessed EMR screening method for the identification of MUPS patients is very specific. However, many patients with MUPS might be missed who scored positive on the PHQ-15, used as a reference test in our dataset. A too stringent search strategy seems the most likely cause. Before using this method, its sensitivity needs to be improved while maintaining its specificity.

## Abbreviations

MUPS: Medically Unexplained Physical Symptoms; GPs: General Practitioners; EMRs: Electronic Medical Records; PHQ-15: Patient Health Questionnaire-15; ICPC: International Classification of Primary Care; PRIME-MD: Primary Care Evaluation of Mental Disorders.

## Competing interests

All authors declare that they have no competing interests.

## Authors’ contributions

All authors made contributions to the research and writing of the manuscript. MB was responsible for planning the study. She also collected, analysed and interpreted the data and wrote the manuscript. TRL and NW developed the EMR screening method. JW, HH and MN were all involved in the conception and design of the study, data analysis and interpretation. All authors supported MB in drafting and revising the manuscript. They all gave their final approval for submission of this version.

## Pre-publication history

The pre-publication history for this paper can be accessed here:

http://www.biomedcentral.com/1471-2296/15/109/prepub

## Supplementary Material

Additional file 1 ICPC codes referring to symptoms suggestive for MUPS. Click here for file
