# Supplementary material for: Identifying patients with medically unexplained physical symptoms in electronic medical records in primary care: a validation study
Source: BMC Fam Pract. 2014 Jun 5;15:109. doi: 10.1186/1471-2296-15-109 (PMC4052805; doi:10.1186/1471-2296-15-109)
Supplement: Additional file 1 — ICPC codes referring to symptoms suggestive for MUPS. [file 1471-2296-15-109-S1.docx]

**ADDITIONAL FILE 1; ICPC codes referring to symptoms suggestive for MUPS**

**Abdomen**

D01 /cramps general

D02 Abdominal pain epigastric

D04 Rectal/anal pain

D06 Abdominal pain localized other

D08 Flatulence/gas/belching

D09 Nausea

D11 Diarrhoea

D12 Constipation

D18 Change faeces/bowel movements

D93 Irritable bowel syndrome

T03 Loss of appetite

T08 Weight loss

**Fatigue**

A04 Weakness/tiredness general

.01 Chronic fatigue syndrome

**Musculoskeletal**

L01 Neck symptom/complaint

L02 Back symptom/complaint

L03 Low back symptom/complaint

L05 Flank symptom/complaint

L06 Axilla symptom/complaint

L07 Jaw symptom/complaint

L08 Shoulder symptom/complaint

L09 Arm symptom/complaint

L10 Elbow symptom/complaint

L11 Wrist symptom/complaint

L12 Hand/finger symptom/complaint

L13 Hip symptom/complaint

L14 Leg/thigh symptom/complaint

L15 Knee symptom/complaint

L16 Ankle symptom/complaint

L17 Foot/toe symptom/complaint

L18 Muscle pain

.01 Fibromyalgia

L79 Sprain/strain of joint NOS

.01 Whiplash trauma cervical spine

**Cardiology-Respiratory**

K01 Heart pain

K02 Pressure/tightness of heart

K03 Cardiovascular pain NOS

K04 Palpitations/awareness of heart

K05 Irregular heartbeat other

L04 Chest symptom/complaint

**(Pseudo-)Neurology and ENT**

A01 Pain general/multiple sites

F13 Eye sensation abnormal

H02 Hearing complaint

H03 Tinnitus, ringing/buzzing ear

N01 Headache

N02 Tension headache

N03 Pain face

N05 Tingling fingers/feet/toes

N17 Vertigo/dizziness

.01 Sensation of unsteadiness

.02 Light-headedness

**Other**

S01 Pruritus

R98 Hyperventilation syndrome

Psychiatry

A26 Fear of cancer NOS

A27 Fear of other disease NOS

B25 Fear of aids/HIV

B26 Fear cancer blood/lymph

B27 Fear blood/lymph disease other

D26 Fear of cancer of digestive system

D27 Fear of digestive disease other

F27 Fear of eye disease

H27 Fear of ear disease

K24 Fear of heart disease

K25 Fear of hypertension

K27 Fear cardiovascular disease other

L26 Fear of cancer musculoskeletal

L27 Fear musculoskeletal disease other

N26 Fear cancer neurological system

N27 Fear of neurological disease other

P01 Feeling anxious/nervous/tense

P06 Sleep disturbance

P75 Somatization disorder

R26 Fear of cancer respiratory system

R27 Fear of respiratory disease other

S26 Fear of cancer of skin

S27 Fear of skin disease other

T26 Fear of cancer of endocrine system

T27 Fear endocrine/metabolic dis other

U26 Fear of cancer of urinary system

U27 Fear of urinary disease other

X23 Fear sexually transmitted disease (f)

X24 Fear of sexual dysfunction female

X25 Fear of genital cancer female

X26 Fear of breast cancer female

Y24 Fear of sexual dysfunction male

Y25 Fear sexually transmitted dis. male

Y26 Fear of genital cancer male

Y27 Fear of genital disease male other

Z29.01 Burnout /stress

Urological/Genital complaints

U02 Urinary frequency/urgency

U05 Urination problems other

X01 Genital pain female

X02 Menstrual pain

X03 Inter menstrual pain

X04 Painful intercourse female

X09 Premenstrual symptom/complaint

X11 Menopausal symptom/complaint

X15 Vaginal symptom/complaint other

X16 Vulvar symptom/complaint

X17 Pelvis symptom/complaint female

Y01 Pain in penis

Y02 Pain in testis/scrotum

Y04 Penis symptom/complaint other

Y08 Sexual function symptom/ complaint (m)
